# Perfectionism and emotional eating among college students: a chained mediation model of body image state and exercise addiction

**DOI:** 10.3389/fpsyg.2026.1840333

**Published:** 2026-05-20

**Authors:** Shuangyan Shi, Shuting Huang, Zhonggen Yin, Qiang Guo, Tong Liu, Fei Dai

**Affiliations:** 1 College of Physical Education, Wuchang Institute of Technology, Wuhan, Hubei, China; 2 Chongqing Yucai Secondary School, Jiulongpo, Chongqing, China; 3 College of Physical Education and Health Management, Chongqing University of Education, Nanan, Chongqing, China; 4 School of Sports Training, Chengdu Sport University, Chengdu, Sichuan, China

**Keywords:** body image state, college student, emotional eating, exercise addiction, perfectionism

## Abstract

**Objective:**

From an integrated perspective combining sociocultural theory and self-determination theory, this study examined whether body image state and exercise addiction sequentially mediate the relationship between perfectionism and emotional eating.

**Methods:**

A cross-sectional design was adopted. A total of 1,307 Chinese college students (aged 18–24 years, *M* = 20.00, SD = 1.18) were recruited using stratified sampling. Standardized scales were used to assess perfectionism, body image state, exercise addiction, and emotional eating. After controlling for demographic variables such as gender and grade, a bias-corrected bootstrap method was employed to test the chained mediation effects.

**Results:**

Correlation analysis revealed a significant positive association between perfectionism and emotional eating. Mediation analysis indicated that perfectionism was associated with emotional eating through three indirect pathways (as indicated by bias-corrected bootstrap 95% confidence intervals that did not contain zero). (a) *via* body image state alone (β = 0.028, p < 0.001); (b) *via* exercise addiction alone (β = 0.022, *p* < 0.001); and (c) *via* the sequential path from body image state to exercise addiction (β = 0.011, *p* < 0.001).

**Conclusion:**

The findings suggest that perfectionism is directly and positively associated with emotional eating among college students, and that this association operates through three indirect pathways, including a sequential pathway *via* body image state and exercise addiction. This pattern is consistent with a sequential psycho-behavioral chain, that is, “personality trait → body-related cognitive evaluation → behavioral pattern → emotional eating.” Given the cross-sectional design, future longitudinal research is needed to establish temporal precedence.

## 1 Introduction

In recent decades, there has been growing concern regarding mental health and adjustment issues among college students. Among these, emotional eating, as an unhealthy coping mechanism, has become a hot topic of research in the field of health psychology (Díaz et al., 2023). Emotional eating refers to the tendency of individuals to relieve discomfort through overeating when facing negative emotions such as anxiety, depression, or stress, rather than being driven by physical hunger (Braden et al., 2025). This behavioral pattern is not only closely linked to physical health issues such as obesity and eating disorders (Chew et al., 2025), but may also exacerbate emotional distress, creating a vicious cycle (Braden et al., 2023, 2025). College students are in a critical transitional phase from adolescence to adulthood, facing multiple pressures such as academic competition, social adjustment, and future planning. Consequently, the prevalence of emotional eating among them is significantly higher than in the general population (Chew et al., 2025). Therefore, in-depth exploration of the factors associated with emotional eating in college students holds significant practical importance for developing effective prevention and intervention strategies.

When examining the psychological factors related to emotional eating, the personality trait of perfectionism has consistently been a focal point of research. The negative emotions and difficulties in emotional regulation associated with perfectionism are key mechanisms underlying disordered eating, including emotional eating (Bernabéu-Brotóns and Marchena-Giráldez, 2022). Perfectionism is typically defined as a stable personality tendency characterized by an individual's pursuit of flawlessness and a tendency to evaluate their own behavior harshly (Frost et al., 1990).

Body image state is considered a key proximal factor linking perfectionism to unhealthy eating behaviors (Rahimi et al., 2025). From the perspective of sociocultural theory, individuals with strong perfectionist tendencies are particularly susceptible to internalizing externally prescribed appearance ideals, which in turn contributes to a negative body image state. Perfectionism is associated with greater body image preoccupation through its link to psychological rigidity, a tendency to avoid and dwell on negative bodily experiences, thereby connecting to unhealthy eating behaviors (Mahboubi et al., 2026). Body image state refers to an individual's perceptions, thoughts, and feelings regarding their physical appearance within a specific temporal and spatial context (Cash, 2002). Individuals with strong perfectionist tendencies often extend rigorous standards to their own appearance, making them highly susceptible to internalizing sociocultural ideals and developing a negative body image state (Hallez et al., 2026). This negative body image state triggers persistent psychological distress and negative emotions (Puri and Sharma, 2025), which may drive individuals to resort to extreme compensatory behaviors. Exercise addiction, characterized by loss of control over exercise, even when injured or unwell (Griffiths, 1997), is one such maladaptive behavior, often motivated by weight or body image control (Bamber et al., 2000). A negative body image state may thus increase the risk of exercise addiction (Edlund et al., 2022).

Exercise addiction may share psychological risk factors such as emotional dysregulation with emotional eating and is highly comorbid among individuals with eating disorders (Amengual-Llofriu et al., 2025). On the one hand, prolonged and excessive compulsive exercise disrupts energy balance, physiologically increasing the drive to eat. To rapidly replenish energy, the body may tend to choose high-calorie foods (Blundell and Beaulieu, 2023). On the other hand, from a psychological perspective, when individuals rely on exercise as a core strategy for regulating emotions and body image, this dependency can trigger a series of negative psychological reactions when exercise plans are disrupted or results fall short of expectations (Gratz and Roemer, 2004). This, in turn, is associated with an increased risk of emotional eating (Kun et al., 2022). Exercise addiction, as a form of behavioral addiction, is also closely associated with difficulties in impulse control and emotional regulation—characteristics that are equally risk factors for emotional eating (Arexis et al., 2023). Therefore, exercise addiction may serve as a link between body image state and emotional eating, translating cognitive evaluations of one's appearance into behavioral patterns, and ultimately relating to disordered eating behaviors.

This study integrates core concepts from sociocultural theory and self-determination theory to construct this chained mediation model. Sociocultural theory emphasizes that sociocultural standards, transmitted through parents, peers, and the media, prompt individuals to internalize external, unrealistic standards of physical perfection (Bolivar, 2020). The process of internalizing these sociocultural appearance standards is a key psychological mechanism associated with negative body image states (Vartanian et al., 2023). In contrast, self-determination theory posits that behavior is driven by external control rather than intrinsic motivation when an individual's fundamental psychological needs, such as autonomy, competence, and relatedness, are thwarted (Ryan and Deci, 2000). A tendency toward perfectionism reflects this sense of external control, driving individuals to act in accordance with external standards rather than to satisfy their internal needs (Almásy et al., 2025). Against this motivational backdrop, individuals become more sensitive to and dissatisfied with their body image state. This may lead them to resort to compensatory, uncontrolled exercise behaviors—that is, exercise addiction. Ultimately, when faced with inevitable setbacks and negative emotions, they may show a stronger association with emotional eating as a maladaptive coping strategy. Sociocultural theory provides a framework for understanding the link between perfectionism and body image state, explaining how external appearance standards are internalized into self-evaluations. Self-determination theory, in turn, offers a motivational explanation for the subsequent links from body image state to exercise addiction and from exercise addiction to emotional eating, highlighting how frustration of basic psychological needs may lead to externally controlled behavioral patterns. Together, these two theories form a complementary framework that addresses different stages of the proposed chain.

In summary, this study aims to explore the associations linking perfectionism and emotional eating among college students. Based on a cross-sectional design, a chained mediation model is constructed to test the sequential mediating roles of body image state and exercise addiction. These three variables constitute a potential pathway through which perfectionism is associated with emotional eating. Theoretically, this study is the first to introduce body image state and exercise addiction as sequential mediators in this field, integrating sociocultural theory with self-determination theory. This integrated perspective helps elucidate how perfectionism is linked to emotional eating through a chain involving cognitive evaluations of body image and behavioral patterns. At the practical level, the findings may provide guidance for mental health interventions among college students. The results suggest that while addressing perfectionist personality traits, efforts could also focus on helping students develop a positive and stable body image state, while maintaining necessary vigilance regarding excessive compulsive exercise behaviors. This approach offers a potential entry point for disrupting maladaptive psychological and behavioral patterns.

## 2 Literature review and hypothesis development

### 2.1 Perfectionism and emotional eating

The relationship between perfectionism and emotional eating has attracted widespread attention from researchers. Existing research has revealed a significant positive association between perfectionism and emotional eating, indicating that perfectionism is one of the key factors associated with this unhealthy eating behavior. This suggests that the link between perfectionism and emotional eating may involve complex pathways, and examining direct effects alone is insufficient to reveal its underlying mechanisms.

Although previous studies have established a positive association between perfectionism and emotional eating, the motivational mechanisms underlying this link remain underexplored. Self-determination theory offers a useful lens, suggesting that perfectionism reflects a pattern of controlled motivation driven by external standards rather than intrinsic needs. This motivational orientation may impair self-regulation, making individuals more vulnerable to emotional eating when faced with negative emotions.

From the perspective of self-determination theory, perfectionist tendencies reflect a state of frustration regarding basic psychological needs. When individuals experience persistent frustration in basic psychological needs such as autonomy, competence, and relatedness, their behavioral motivation shifts from internal drive to external control; that is, they act to conform to external standards rather than to satisfy internal needs (Klein, 2019). Under this motivational framework, when faced with negative emotions, individuals may lack effective internal regulatory resources and resort to emotional eating, a maladaptive coping strategy, to seek immediate emotional relief. This theoretical perspective provides a deep motivational foundation for understanding the association between perfectionism and emotional eating, suggesting that perfectionism may be associated with greater reliance on emotional eating through its link to impaired self-regulation.

Based on the aforementioned theoretical and empirical foundations, this study proposes the following hypothesis:

*Hypothesis 1*: Perfectionism is positively associated with emotional eating.

### 2.2 Perfectionism, body image state, and emotional eating

Body image state is considered a key factor linking perfectionism to unhealthy eating behaviors (Martin et al., 2025). It refers to an individual's perceptions, thoughts, and feelings regarding their own physical appearance within a specific temporal and spatial context (Cash, 2002). Individuals with strong perfectionist tendencies often extend their strict evaluative standards to their own appearance, making them highly susceptible to developing a negative body image state (Szymajda et al., 2026). Existing research has confirmed a significant positive association between perfectionism and body image dissatisfaction: the higher the perfectionist tendency, the more pronounced the negative evaluation of one's own body image (Ahvan et al., 2025).

Sociocultural theory offers a parsimonious explanation for this link, suggesting that individuals internalize externally prescribed appearance ideals, transmitted through parents, peers, and media, as self-imposed standards (Rodgers et al., 2020). Perfectionism amplifies this internalization process, increasing sensitivity to social pressures and thereby contributing to body image dissatisfaction (Ananta and Suhadianto, 2022). A negative body image state, in turn, serves as a source of psychological distress, triggering negative emotions. To regulate these unpleasant emotional experiences, individuals may resort to emotional eating as a coping strategy (Wang et al., 2025). Previous research among college students has consistently shown a significant association between body image dissatisfaction and disordered eating behaviors, including emotional eating (Jiménez-Limas et al., 2022). Based on the above theoretical and empirical evidence, this study proposes the following hypothesis:

*Hypothesis 2*: Body image state mediates the relationship between perfectionism and emotional eating.

### 2.3 Perfectionism, exercise addiction, and emotional eating

Exercise addiction is a maladaptive behavior that has garnered attention in the field of health psychology in recent years (Macía et al., 2025). While moderate exercise is beneficial to health (Gonzalez-Santamaria et al., 2025), exercise addiction manifests as a loss of control over physical activity (Griffiths, 1997). Individuals engage in compulsive exercise even when injured or unwell, and the core motivation is often closely linked to weight or body image control (Bamber et al., 2000).

It is important to distinguish exercise addiction from mere compulsive or problematic exercise. While compulsive exercise refers to an uncontrolled urge to exercise driven by a sense of duty or guilt, exercise addiction is a broader behavioral addiction characterized by a set of core components: salience (exercise dominates the individual's life), conflict (exercise causes interpersonal or intrapersonal conflicts), emotional regulation (exercise is used to modify mood states), tolerance (increasing amounts of exercise are needed to achieve the same effect), withdrawal (negative emotional states when unable to exercise), and relapse (return to excessive exercise after attempts to reduce; Griffiths, 1997). The present study adopts this multidimensional conceptualization, as operationalized by the Exercise Addiction Inventory (EAI). Thus, the term “exercise addiction” throughout this manuscript refers to the full syndrome, not merely the compulsive aspect.

Individuals with strong perfectionist tendencies, due to their strict standards regarding personal performance and outward appearance, may view exercise as a key means to achieve their ideal body shape (Berki et al., 2025). This perspective may increase the risk of excessive engagement and addiction. Research has found that perfectionism is associated with an “obsessive passion” for exercise, and this pattern is also related to higher levels of eating disorders (St-Cyr et al., 2024). This finding suggests a positive association between perfectionism and exercise addiction.

Exercise addiction may be associated with emotional eating through both physiological and psychological pathways. Physiologically, excessive exercise disrupts energy balance, increasing cravings for high-calorie foods (Beaulieu et al., 2020). Psychologically, when individuals rely on exercise as a primary emotion regulation strategy, disruptions to their exercise routine may trigger negative emotions, which in turn increase the risk of emotional eating (Meyer et al., 2011).

From the perspective of self-determination theory, when an individual's basic psychological needs are thwarted, they experience “need frustration.” This experience significantly alters their behavioral drive patterns, shifting them from being driven by intrinsic motivation, which derives from interest, enjoyment, and intrinsic value, to being driven by controlled motivation (Howard et al., 2025). Perfectionists are more likely to be driven by external expectations or internal pressures rather than by intrinsic interest or value (Frost et al., 1990).

Against this motivational backdrop, individuals may resort to compulsive, compensatory exercise in an attempt to control their body image state or as an alternative means of emotional regulation (Meyer et al., 2011). However, as a behavioral addiction, exercise addiction is closely linked to difficulties in impulse control and emotional regulation (El Archi et al., 2022). When individuals rely on exercise to regulate their emotions, they are highly susceptible to feelings of frustration and a sense of loss of control if their exercise plans are disrupted or fail to achieve the desired body shaping results (Meyer et al., 2011). This negative emotional state, in turn, is associated with emotional eating.

Based on the aforementioned theoretical and empirical evidence, this study proposes the following hypothesis:

*Hypothesis 3*: Exercise addiction mediates the relationship between perfectionism and emotional eating.

### 2.4 Perfectionism, body image state, exercise addiction, and emotional eating: a chained mediation pathway

The preceding sections have separately discussed the independent mediating roles of body image state and exercise addiction in the relationship between perfectionism and emotional eating. However, these two mediating variables are not isolated from one another. Rather, there may be a logical sequential relationship between them, together forming a sequential mediation pathway. To explore the validity of this chained pathway, it is crucial to clarify the intrinsic connection between body image state and exercise addiction.

A theoretical logical link exists between body image state and exercise addiction. When individuals hold negative perceptions and evaluations of their own body image, this experience is often accompanied by a strong desire to alter their physical appearance (Su et al., 2025). Given its role in shaping body composition, exercise naturally becomes the individual's preferred compensatory mechanism (Cook et al., 2016). When exercise behavior is primarily driven by external standards and pressures rather than intrinsic enjoyment or value alignment, which refers to autonomous motivation, individuals are more likely to experience a loss of behavioral control and compulsive traits (Özkan, 2025). This pattern is associated with a higher risk of exercise addiction.

From the perspective of self-determination theory, when individuals experience persistent frustration of basic psychological needs due to a negative body image state, their behavioral motivation is more likely to be dominated by external control. This means they may seek external validation or attempt to escape internal distress through compulsive exercise. This motivational context is closely aligned with the theoretical mechanisms associated with the development of exercise addiction (Savvador et al., 2026). Existing research provides preliminary empirical evidence for this. For example, a study of regular exercise participants in Lebanon confirmed that body appreciation is associated with the relationship between exercise addiction and eating disorders. The lower the level of body appreciation, the stronger the positive association between exercise addiction and eating disorders (Abi Karam et al., 2025).

Although it is theoretically plausible that exercise addiction could precede negative body image, existing evidence suggests that body-related cognitive evaluations typically serve as antecedents rather than consequences of compulsive exercise behaviors. Accordingly, the proposed order, namely body image state followed by exercise addiction, is more consistent with the cognitive-behavioral sequence underlying these phenomena.

Based on the above analysis, perfectionism may be associated with emotional eating through a sequential transmission involving body image state and exercise addiction. The logical connection in this chain of pathways can be explained as follows. First, perfectionism, as a stable personality trait, is positively associated with an individual's negative cognitive evaluation of their body image. Second, a negative body image state is positively associated with a tendency toward exercise addiction. That is, the higher the negative evaluation of body image, the higher the risk of compulsive exercise behavior. Third, exercise addiction is also positively associated with emotional eating. That is, the stronger the tendency toward exercise addiction, the more frequently emotional eating may occur. These three links connect sequentially, forming a potential psycho behavioral chain that extends from personality traits to body cognition, then to behavioral patterns, and ultimately to eating behaviors.

**Figure 1:**
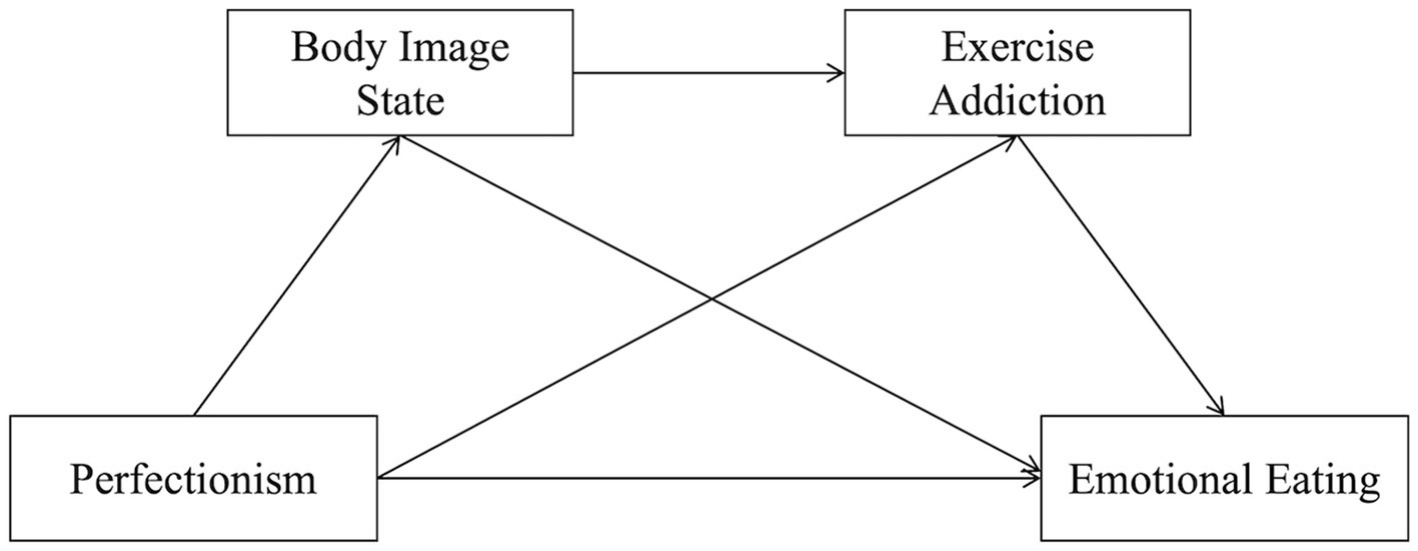
The chain mediation effect model.

Based on the aforementioned theoretical analysis and logical reasoning, this study proposes the following core hypothesis:

*Hypothesis 4*: Body image state and exercise addiction are associated with the relationship between perfectionism and emotional eating in a sequential manner. Specifically, perfectionism is positively associated with negative body image state; negative body image state is positively associated with exercise addiction; and exercise addiction is positively associated with emotional eating. These three variables constitute a potential sequential pathway linking perfectionism and emotional eating (Figure 1).

## 3 Methodology

### 3.1 Procedures and participants

This study employed a cross-sectional design. Data collection took place between November 1 and November 30, 2025. Using stratified sampling, a total of 1,400 adult college students were recruited from 15 provinces across five regions of China: eastern, central, western, southern, and northern.

Class advisors were responsible for distributing the questionnaires. Prior to distribution, the research team provided specialized training to the teachers. The training covered key aspects of questionnaire administration, including the voluntary participation process, opt out mechanisms, and confidentiality requirements. Subsequently, class advisors distributed the questionnaires to the college students. Participants completed the questionnaires under the supervision of their class advisors. During this process, teachers provided detailed explanations of the principle of voluntary participation, the meaning of each item, and the respondents' right to withdraw.

The final sample included 1,307 participants. Their ages ranged from 18 to 24 years, with a mean age of 20 years (SD = 1.175). Among them, 627 were male (47.97%) and 680 were female (52.03%). The distribution by academic year was as follows: 435 first year students (33.28%), 456 second year students (34.89%), 308 third year students (23.57%), and 108 fourth year students (8.26%). In terms of household registration, 598 participants were urban residents (45.75%) and 709 were rural residents (54.25%). The effective response rate was 93.36%.

Exclusion criteria included: (a) severe physical illness; (b) history of mental illness; (c) receipt of psychological treatment within the past 3 months; (d) response time that was significantly too short or too long; and (e) invalid response patterns, such as repeated answers or internal contradictions.

Data were collected *via* an online platform (https://www.wenjuan.com/s/UZBZJvYWmq/#) using an electronic questionnaire. The questionnaire comprised five sections: basic information (grade level, gender, height, and weight), the Perfectionism Scale, the Body Image State Scale, the Exercise Addiction Risk Scale, and the Emotional Eating Scale.

After data collection, all information was anonymized to ensure confidentiality. The anonymized dataset will be securely stored in accordance with institutional ethical guidelines and data management policies. It will remain available for research purposes for at least 5 years following publication. Qualified researchers may request access to the data, provided they sign a formal data sharing agreement to ensure participant privacy and comply with the original informed consent terms.

### 3.2 Ethics approval and consent to participate

This study was conducted in strict accordance with the ethical guidelines of the Declaration of Helsinki. The study protocol was approved by the Ethics Committee of Chengdu Sport University (Approval No.: CTYLL2025202).

Prior to formal participation, all participants signed an electronic informed consent form *via* an online platform. The informed consent form detailed the study objectives, the principle of voluntary participation, confidentiality agreements, and the right to withdraw from the study at any time without incurring any liability.

To protect participant privacy, data were anonymized during collection and analysis. No personally identifiable information was retained in the final dataset.

### 3.3 Measurement tools

#### 3.3.1 Perfectionism

This study used the Frost Multidimensional Perfectionism Scale (FMPS) to assess perfectionism traits (Frost et al., 1990). The scale consists of 27 items across five dimensions. Sample items include: “Being organized and systematic is very important to me” and “I try to be an organized person.” Items were scored on a 5 point Likert scale ranging from 1 (strongly disagree) to 5 (strongly agree). Higher total scores indicated stronger perfectionism traits. Confirmatory factor analysis was conducted to evaluate the construct validity of the scale. Model fit indices indicated good fit: χ^2^/df = 1.330, GFI = 0.977, AGFI = 0.972, RMSEA = 0.016. The Cronbach's α coefficient for this scale in the present study was 0.905.

#### 3.3.2 Body image state scale

Body image state was measured using the Body Image States Scale (BISS), developed by Cash et al. (2002). This single dimensional scale consists of six items. A sample item is: “How satisfied are you with your appearance?” Responses were recorded on a 9 point Likert scale ranging from 1 (very dissatisfied) to 9 (very satisfied). Higher total scores indicated a more positive body image state. Confirmatory factor analysis was conducted to evaluate the construct validity of the scale. Model fit indices indicated good fit: χ^2^/df = 0.971, GFI = 0.998, AGFI = 0.995, RMSEA = 0.000. The Cronbach's α coefficient for this scale in the present study was 0.892.

#### 3.3.3 Exercise addiction scale

This study used the Exercise Addiction Inventory (EAI) to measure the risk of exercise addiction (Griffiths et al., 2005). The scale consists of six items assessing six dimensions: salience, conflict, emotional regulation, tolerance, withdrawal symptoms, and relapse. Responses were recorded on a 5 point Likert scale ranging from 1 (strongly disagree) to 5 (strongly agree).

The EAI measures the addictive symptoms and tendencies individuals exhibit in their exercise behavior. Higher scores on the scale indicate a higher risk of exercise addiction, rather than a clinical diagnosis. Confirmatory factor analysis was conducted to evaluate the construct validity of the scale. Model fit indices indicated good fit: χ^2^/df = 5.756, GFI = 0.987, AGFI = 0.969, RMSEA = 0.060. The Cronbach's α coefficient for this scale in the present study was 0.908.

#### 3.3.4 Emotional eating scale

Emotional eating was assessed using a subscale from the Dutch Eating Behavior Questionnaire (DEBQ), developed by Van Strien et al. (1986). This subscale was used to evaluate emotional eating behaviors among college students. It consists of 13 items measuring eating behaviors under emotional states such as anger, boredom, disappointment, or tension. A sample item is: “Do you have the desire to eat when you are irritated?”.

Responses were recorded on a 5 point Likert scale ranging from 1 (never) to 5 (always). Higher scores indicated more frequent emotional eating behaviors. Confirmatory factor analysis was conducted to evaluate the construct validity of the scale. Model fit indices indicated good fit: χ^2^/df = 3.637, GFI = 0.972, AGFI = 0.961, RMSEA = 0.045. The Cronbach's α coefficient for internal consistency in this study was 0.932.

### 3.4 Data analysis

The data analysis procedure consisted of three steps.

First, common method bias was assessed using Harman 's one factor test.

Second, descriptive statistics and correlation analysis were performed. Means, standard deviations, and Pearson correlation coefficients were calculated for all variables, including perfectionism, body image state, exercise addiction, and emotional eating.

Third, a chain mediation analysis was conducted. The PROCESS macro (version 4.0) in SPSS 25.0 was used to construct a chain mediation model. This model examined the pathway from perfectionism to emotional eating through the mediating effects of body image state and exercise addiction. Gender, grade level, and body mass index were included as covariates in the analysis. Indirect effects were tested using the bootstrap resampling method with 5,000 iterations and 99% confidence intervals.

## 4 Results

### 4.1 Common method bias test

Because the questionnaire data were obtained through self-report by the participants, Harman's single factor test was used to assess common method bias. This test was conducted to ensure that the research findings were not significantly affected by such bias.

Exploratory factor analysis was performed on all items from the Perfectionism Scale, the Body Image State Scale, the Exercise Addiction Inventory, and the Emotional Eating Scale. Principal component analysis was used to extract common factors, and partial correlations were calculated based on the first common factor.

The results showed that eight factors had eigenvalues greater than 1. The first factor explained 19.724% of the variance, which was below the 40% critical threshold. This indicates that common method bias was not a significant concern in this study.

### 4.2 Descriptive statistics and correlations

Descriptive statistics and Pearson correlation analyses were calculated for the four variables: perfectionism, body image state, exercise addiction, and emotional eating (Table 1). All variables showed significant interrelationships.

**Table 1:** Descriptive statistics and correlation analysis of variables.

Variable	M	SD	Perfectionism	Body image state	Exercise addiction	Emotional eating
Perfectionism	88.678	13.833	1			
Body image state	29.396	8.954	−0.244^**^	1		
Exercise addiction	19.565	4.826	0.272^**^	−0.413^**^	1	
Emotional eating	42.562	9.186	0.185^**^	−0.194^**^	0.201^**^	1

^**^p < 0.01.

Specifically, Perfectionism was positively correlated with emotional eating (*r* = 0.185, *p* < 0.01); Perfectionism was negatively correlated with body image state (*r* = −22120.244, *p* < 0.01); Perfectionism was positively correlated with exercise addiction (*r* = 0.272, *p* < 0.01); Body image state was negatively correlated with exercise addiction (*r* = −0.413, *p* < 0.01); Body image state was negatively correlated with emotional eating (*r* = −0.194, *p* < 0.01); Exercise addiction was positively correlated with emotional eating (*r* = 0.201, *p* < 0.01).

### 4.3 Chain-mediated effects analysis

To examine the associations of perfectionism, body image state, and exercise addiction with emotional eating, a hierarchical regression analysis was conducted. Perfectionism, body image state, and exercise addiction were entered as independent variables, with emotional eating as the dependent variable (Table 2).

**Table 2:** Regression analysis of perfectionism, body image state, and exercise addiction on emotional eating.

Variant	Emotional eating			
	β	*T*	*F*	*R* ^2^
Perfectionism	0.185	4.413^***^	46.011	0.033
Body image state	−0.194	−7.140^***^	50.977	0.037
Exercise addiction	0.201	7.413^***^	54.955	0.040

^***^p < 0.001.

The results showed that: Perfectionism was positively associated with emotional eating (β = 0.185, *t* = 4.413, *p* < 0.001); Body image state was negatively associated with emotional eating (β = −0.184, *t* = −7.140, *p* < 0.001); Exercise addiction was positively associated with emotional eating (β = 0.201, *t* = 7.413, *p* < 0.001). After controlling for gender, grade level, and BMI, the chained mediation model remained significant, indicating the stability of the findings.

This study examined the chain mediation effects of body image state and exercise addiction on the relationship between perfectionism and emotional eating. Perfectionism (X) was the independent variable, emotional eating (Y) was the dependent variable, and body image state (W1) and exercise addiction (W2) were the mediating variables. Demographic variables (gender and grade level) were included as covariates.

Analysis was conducted using the PROCESS macro (version 3.4) in SPSS 25.0 to test whether perfectionism was associated with emotional eating through significant mediating and chain mediating effects. Hypothesis testing was performed using Model 6 of the PROCESS macro, and the chain mediation effects were verified using 5,000 bootstrap samples and 95% confidence intervals (CIs).

As shown in Table 3, perfectionism was significantly associated with emotional eating among college students [β = 0.185, *p* < 0.001; 95% CI (0.131, 0.238)]. This result supported Hypothesis 1.

**Table 3:** Mediation effect values and effect sizes.

Effect pathway	Effect	BOOT SE	BOOT LLCI	BOOT ULCI	Relative mediation effect (%)
Total effect	0.185^***^	0.027	0.131	0.238	100
Direct effect	0.124^***^	0.028	0.069	0.179	67.027
Total indirect effect	0.061^***^	0.011	0.040	0.083	32.973
Indirect effect 1 (body image state)	0.028^***^	0.008	0.013	0.044	15.135
Indirect effect 2 (exercise addiction)	0.022^***^	0.006	0.010	0.036	11.892
Indirect effect 3 (body image state and exercise addiction)	0.011^***^	0.003	0.005	0.018	5.946

Indirect effect 1: perfectionism → body image state → emotional eating.

Indirect effect 2: perfectionism → exercise addiction → emotional eating.

Indirect effect 3: perfectionism → body image state → exercise addiction → emotional eating.

^***^p < 0.001.

The relationship between perfectionism and emotional eating was mediated by body image state [*p* < 0.001; 95% CI (0.013, 0.044)], suggesting that perfectionism was linked to emotional eating through body image state. This finding supported Hypothesis 2.

Exercise addiction also mediated the association between perfectionism and emotional eating [*p* < 0.001; 95% CI (0.010, 0.036)], indicating that perfectionism was linked to emotional eating through exercise addiction. This finding supported Hypothesis 3.

Finally, body image state and exercise addiction sequentially mediated the relationship between perfectionism and emotional eating [*p* < 0.001; 95% CI (0.005, 0.018)]. This demonstrated that perfectionism was associated with emotional eating through a chained pathway involving body image state and exercise addiction, thereby supporting Hypothesis 4.

The total effect, direct effect, and all three indirect effects were statistically significant (*p* < 0.001). The total effect size was 0.185. The direct effect was 0.124, accounting for 66.03% of the total effect. The total indirect effect was 0.061, accounting for 32.97% of the total effect.

**Figure 2:**
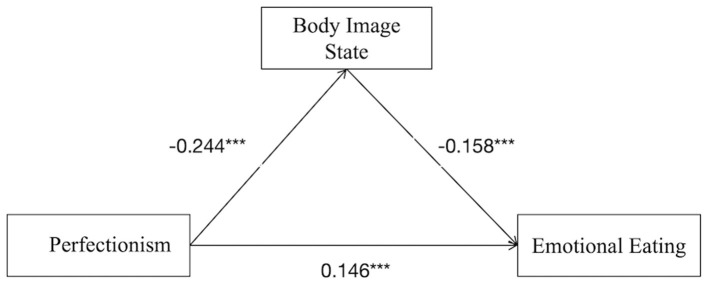
Model of mediating roles of body image state between perfectionism and emotional eating.

**Figure 3:**
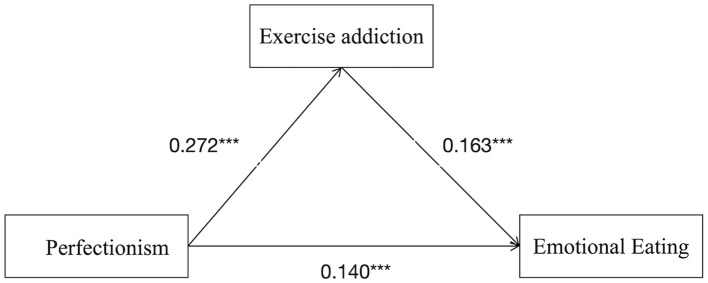
Model of mediating roles of exercise addiction between perfectionism and emotional eating.

**Figure 4:**
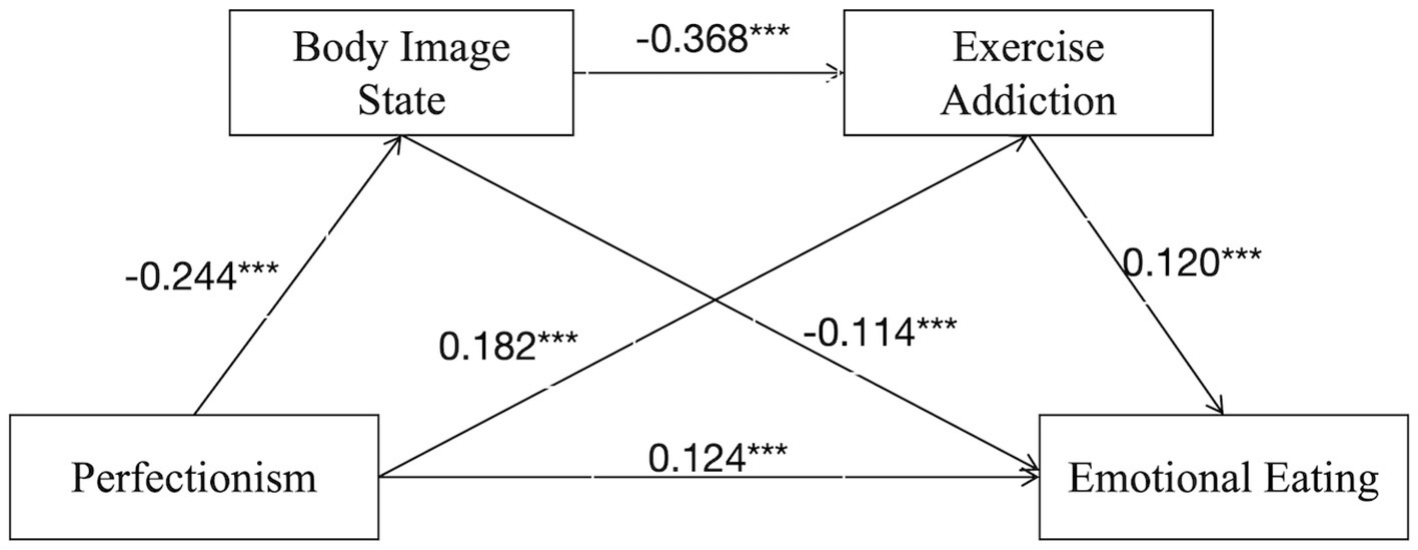
Model of chain mediating roles of body image state and exercise addiction between perfectionism and emotional eating.

Among the three indirect pathways: Path 1 (perfectionism → body image state → emotional eating): effect size = 0.028, accounting for 15.14% of the total indirect effect; Path 2 (perfectionism → exercise addiction → emotional eating): effect size = 0.022, accounting for 11.89% of the total indirect effect; Path 3 (perfectionism → body image state → exercise addiction → emotional eating): effect size = 0.011, accounting for 5.95% of the total indirect effect. The specific pathways are illustrated in Figure 2 (mediation *via* body image state), Figure 3 (mediation *via* exercise addiction), Figure 4 (chained mediation).

## 5 Discussion

This study aimed to explore the mechanisms underlying the association between perfectionism and emotional eating among college students, with a particular focus on the chained mediating roles of body image state and exercise addiction. The results were consistent with the initial theoretical hypotheses, revealing that perfectionism was not only directly and positively associated with emotional eating but was also linked to it through three indirect pathways: a single mediation pathway *via* body image state, a single mediation pathway *via* exercise addiction, and a sequential mediation pathway involving both body image state and exercise addiction. This finding reveals multiple channels through which perfectionism and emotional eating are associated. The establishment of the sequential mediation pathway is particularly noteworthy, as it provides a new integrative perspective for understanding how perfectionism ultimately relates to emotional eating through the sequential transmission of body related cognitive evaluations and behavioral patterns.

### 5.1 Main findings

This study found a significant positive association between perfectionism and emotional eating, a result consistent with previous research. Previous studies have confirmed that individuals with strong perfectionistic tendencies tend to use emotional eating as a coping strategy when facing negative emotions (Mamo et al., 2024). As highlighted in Fekih-Romdhane et al.'s (2024) cross-national study (2024) and the meta-analysis by Grant and Chamberlain (2023), perfectionism is associated with eating disorders. This study reaffirmed the stability of this association within a college student population, extending existing findings from clinical samples to the general college student population. It also provided empirical support from a Chinese college student sample for perfectionism as a factor associated with emotional eating.

Regarding the mediating role of body image state, this study found that body image state partially mediated the relationship between perfectionism and emotional eating. This result is consistent with sociocultural theory, which posits that perfectionist tendencies are associated with greater sensitivity to socially and culturally transmitted ideal body standards. This sensitivity makes individuals more likely to internalize these standards as self-imposed demands, which in turn is associated with a negative body image state. A negative body image state, as a persistent source of psychological distress, is positively associated with the occurrence of emotional eating (Castro-López et al., 2023; Szymajda et al., 2026). Previous studies, such as Guo et al. series on sociocultural pressures and eating disorders (Guo et al., 2023), as well as Behrend et al. exploration of the relationship between body appreciation and intuitive eating (Behrend et al., 2024), have all highlighted the mediating role of body image state between external pressures and eating behaviors. Building on this foundation, the present study further deepened the analysis by introducing body image state into the framework linking perfectionist personality traits and emotional eating. It revealed that body image state is not only a factor directly associated with eating problems but also a key explanatory mechanism for the relationship between perfectionist personality traits and emotional eating, thereby expanding the application of sociocultural theory in the field of personality and eating relationships.

Regarding the mediating role of exercise addiction, this study found that exercise addiction similarly played a partial mediating role between perfectionism and emotional eating. This result can be understood from the perspective of self-determination theory. Previous surveys of athletes have found that individuals with higher perfectionist tendencies also have a correspondingly higher risk of exercise addiction and often exhibit unhealthy eating attitudes (Berki et al., 2025). However, those studies did not further examine the internal pathways among the three variables. Another study grounded in self-determination theory suggested that the degree to which basic psychological needs are met is closely related to the autonomy of exercise behavior (Hosseini et al., 2022). Building upon these studies, the present research incorporated exercise addiction as a mediating variable into the relationship model between perfectionism and emotional eating. It revealed that perfectionism may be associated with emotional eating through the behavioral pathway of exercise addiction, providing a new behavioral level explanatory mechanism for understanding how perfectionism is associated with eating behaviors. This addressed a limitation of previous research, which often focused on cognitive and emotional factors while paying insufficient attention to behavioral mediation.

This study also found that body image state and exercise addiction constituted a complete sequential transmission pathway between perfectionism and emotional eating. This result implies that perfectionism is associated with emotional eating not only through the individual channels of body image state or exercise addiction, but also through the chain pathway of

“perfectionism → negative body image state → increased tendency toward exercise addiction → emotional eating.”

The establishment of this pathway revealed the intrinsic logical connection between the two mediating variables. Previous studies have often examined body image state and exercise behavior as parallel or independent mediating factors. For example, Meyer et al. found that body image distress and compulsive exercise are both associated with eating disorders, but the specific pathways linking these variables were not explicitly tested (Meyer et al., 2011). Building on this foundation, the present study further elucidated a potential sequential relationship between the two, suggesting a progressive association between body related cognitive evaluations and behavioral patterns. Together, these elements form a psycho behavioral chain linking personality traits to eating problems, thereby providing a more nuanced perspective for understanding the complex mechanisms underlying the association between perfectionism and emotional eating.

### 5.2 Theoretical contributions and implications

This study makes three primary theoretical contributions.

First, it identifies a sequential order between body image state and exercise addiction. While previous research has largely examined body image and exercise behavior as parallel or co-occurring factors in relation to eating disorders (Meyer et al., 2011; Zou et al., 2022), this study demonstrates that body-related cognitive evaluations precede behavioral patterns. Specifically, perfectionism is associated with a negative body image state, which in turn is associated with exercise addiction, and ultimately linked to emotional eating. This sequence aligns with the fundamental logic of cognitive-behavioral therapy, cognition precedes behavior, and extends prior work by clarifying the temporal order of these two mediators, which previous studies have not explicitly addressed.

Second, it integrates sociocultural theory and self-determination theory into a unified explanatory framework. Sociocultural theory explains the first stage of the chain, elucidating how perfectionist individuals internalize externally prescribed appearance ideals, leading to a negative body image state (Rodgers et al., 2020). Self-determination theory accounts for the subsequent stages, explaining how frustration of basic psychological needs gives rise to externally controlled behavioral patterns—namely exercise addiction and emotional eating (Ryan and Deci, 2000). Although both theories have been independently applied to research on eating behaviors and exercise, few studies have integrated them into a single model. By using body image state as the connecting node, this study offers a comprehensive framework that bridges sociocultural and motivational perspectives, addressing different stages of the psycho-behavioral chain.

Third, it provides a more nuanced explanation for inconsistencies in prior research. Previous studies examining the relationship between perfectionism and emotional eating have reported inconsistent effect sizes or unstable mediating effects (Bernabéu-Brotóns and Marchena-Giráldez, 2022). The chained mediation model proposed in this study suggests that such inconsistencies may arise from oversimplified models that overlook the sequential interplay between body image state and exercise addiction. By capturing the cumulative associations operating through the full psycho-behavioral chain, this study offers a more complete picture of the mechanisms linking perfectionism to emotional eating, and may help explain why interventions targeting single factors have shown limited effectiveness, aligning with prior calls for multi-component intervention approaches (Egan et al., 2011).

### 5.3 Practical significance and application prospects

The chained associations identified in this study provide a multi-level, phased framework for preventing emotional eating and promoting mental health among college students. The complete psycho-behavioral chain—personality traits → body image evaluation → behavioral patterns → eating behaviors—offers distinct entry points for intervention.

At the personality level, interventions can focus on identifying perfectionist tendencies and raising awareness of their potential links to body image and behavioral outcomes. Rather than aiming to fundamentally alter perfectionism, a relatively stable trait, efforts can be directed toward cultivating more flexible self-evaluation standards and mitigating the negative influence of perfectionism on body image state.

At the body image level, practical strategies include media literacy education to help students critically examine sociocultural appearance ideals, and body acceptance training that emphasizes functionality over appearance. Improving body image state may reduce both the risk of emotional eating and the likelihood of progressing toward exercise addiction.

At the behavioral transition stage, educators in physical education and fitness settings can promote healthy exercise principles, helping students distinguish between moderate and compulsive exercise. Fostering exercise motivations centered on enjoyment and health, rather than body transformation as the sole goal, may prevent the shift from cognitive distress to behavioral addiction.

At the later stages, interventions should focus on cultivating emotional regulation skills and expanding adaptive coping strategies. For students already showing signs of exercise addiction or emotional eating, early identification and professional referral channels are essential to prevent further escalation.

### 5.4 Research limitations and future directions

This study has several limitations that should be considered when interpreting the findings.

First, regarding study design, this study employed a cross-sectional design, which precludes causal inferences. The findings reflect covariation and associative relationships among variables, and no conclusions regarding temporal sequence or causality can be drawn. The sequential pathways proposed in the chained mediation model were primarily derived from theoretical reasoning; their directionality requires further validation through longitudinal or experimental designs.

Second, regarding model specification, this study tested the chained mediation effects of body image state and exercise addiction. However, other mediating variables (e.g., emotion regulation, self-esteem) or alternative model specifications (e.g., bidirectional relationships between body image and exercise addiction) may exist and warrant further exploration.

Third, regarding boundary conditions, this study did not examine potential moderators of the chain mediation pathways. Future research could incorporate individual-level variables (e.g., gender, grade level, and BMI) or contextual factors (e.g., social support, cultural context) to test moderated mediation models, thereby identifying the conditions under which the chain pathway operates.

Fourth, regarding sample representativeness, this study focused on college students aged 18–24 years recruited from 15 provinces across five regions of China. While this provides broad geographic coverage, caution is warranted when generalizing the findings to other populations (e.g., adolescents, clinical populations, and non-Chinese cultural contexts). Future research should examine the generalizability of the chain mediation model across diverse age groups and cultural settings.

Fifth, regarding measurement tools, this study relied on self-report questionnaires, which may be subject to recall bias and social desirability bias. Future research could incorporate multi-source data (e.g., informant reports, behavioral observations) or employ ecological momentary assessment to capture real-time fluctuations in body image state, exercise behavior, and emotional eating in naturalistic settings.

Sixth, this study treated perfectionism as a global construct without distinguishing between adaptive and maladaptive dimensions. Adaptive perfectionism (e.g., personal standards) may be associated with healthier psychological outcomes, whereas maladaptive perfectionism (e.g., concern over mistakes) is a risk factor for emotional eating. Future research could explore whether the chained mediation model operates differently across these two dimensions. Additionally, interventions aimed at fostering adaptive perfectionism, such as promoting self-oriented striving rather than socially prescribed perfectionism, warrant investigation as potential preventive strategies for emotional eating.

## 6 Conclusion

Drawing on sociocultural theory and self-determination theory, this study examined the chained mediation model linking perfectionism to emotional eating among Chinese college students. The results revealed that perfectionism was directly and positively associated with emotional eating. Moreover, this association operated through three indirect pathways: *via* body image state alone, *via* exercise addiction alone, and sequentially *via* body image state followed by exercise addiction.

These findings suggest a psycho-behavioral chain extending from personality traits to body-related cognitive evaluations, then to behavioral patterns, and ultimately to emotional eating. By integrating sociocultural theory and self-determination theory across different stages of this chain, the study offers a unified framework for understanding how perfectionism is associated with emotional eating through multivariate sequential transmission. Practically, the findings highlight multiple intervention entry points, namely personality, body image, exercise behavior, and emotion regulation, for preventing emotional eating among college students.

Given the cross-sectional design, causal inferences cannot be drawn. Future longitudinal research is warranted to establish temporal precedence and to examine the generalizability of the chained mediation model across diverse populations.

## Data Availability

The original contributions presented in the study are included in the article/supplementary material, further inquiries can be directed to the corresponding author.
